# Patterns of Idiopathic Interstitial Pneumonia and Their Correlation With Spirometry Findings: A Study at Tikur Anbessa Specialized Hospital, Addis Ababa, Ethiopia

**DOI:** 10.1155/rrp/8888453

**Published:** 2025-08-28

**Authors:** Natnael Alemu Bezabih, Michael Teklehaimanot Abera, Henok Dessalegn Damtew, Raja Tamiru Muleta, Bezawit Alemu Bezabih, Abubeker Fedlu Abdela, Selam Muluneh Adane, Azemera Gissila Aboye, Yimaj Abdulwahab, Amir Alwan

**Affiliations:** ^1^Department of Radiology, College of Health Sciences, Addis Ababa University, Addis Ababa, Ethiopia; ^2^School of Medicine, College of Health Sciences, Addis Ababa University, Addis Ababa, Ethiopia; ^3^Faculty of Medicine, College of Public Health and Medical Sciences, Jimma University, Jimma, Ethiopia

## Abstract

**Background:** Idiopathic interstitial pneumonias (IIPs) are widespread interstitial lung diseases with no known cause. The diseases are characterized by a steady decline in lung function.

**Objective:** To assess the patterns of IIPs and investigate the correlation between the extents of lung involvement on high-resolution computed tomography (HRCT) with spirometric findings in Tikur Anbessa Specialized Hospital (TASH) chest clinic, covering the period from February 2020 to February 2023.

**Methods:** An institution-based retrospective, descriptive, and cross-sectional study design was used. This study included all cases diagnosed with IIPs that underwent HRCT and spirometry within a 3-month window. Finally, we used the Pearson correlation test with a 2-tailed significance of less than 0.05 as a cutoff. Then the information is presented using simple frequencies, summary measures, tables, and figures.

**Result:** There were 54 patients diagnosed with IIP. The overall median age of the patients was 53.9 ± 15.4. Nonspecific interstitial pneumonia (NSIP) was the most common interstitial lung disease diagnosed. Ground-glass opacity (GGO) was the most dominant HRCT feature identified. Pearson correlation tests (*p* < 0.05) were used to examine the correlation between lung involvement and spirometry parameters, as well as pulse oximetry–measured oxygen saturation. All spirometry parameters, forced vital capacity (FVC), forced expiratory volume at 1 min (FEV1), peak expiratory flow (PEF), forced expiratory flow (FEF) 25%–75%, and oxygen saturation, demonstrated a significant negative correlation with lung involvement. The strongest correlations were observed with FVC (*r* = −0.827) and FEV1 (*r* = −0.789), both with *p* < 0.001. Oxygen saturation showed a moderate correlation (*r* = −0.49, *p* < 0.001), while PEF and FEF 25%–75% exhibited weak correlations (*r* = −0.39, *p*=0.003, and *r* = −0.38, *p*=0.005, respectively).

**Conclusion and Recommendations:** There was a significant negative correlation between FVC and FEV1 and the extent of lung involvement identified by HRCT in IIPs suggesting a pivotal role of pulmonary function tests (PFTs), specifically FVC, in monitoring IIP progression, supported by HRCT for diagnostic clarity. To enhance IIP patient care, routine PFTs, particularly FVC, are recommended for monitoring.

## 1. Introduction

Idiopathic interstitial pneumonia (IIP) is a diverse category of chronic, progressive, fibrosing, interstitial, and nonneoplastic disorders caused by various inflammation patterns and fibrosis in the lung parenchyma [1, 2]. There have been several modalities used to follow patients with this condition, but high-resolution computed tomography (HRCT) has been employed in the assessment of these patients to establish safe, cost-effective parameters to evaluate disease progression [1]. HRCT, by describing the pattern and distribution of features that closely correlate with histological findings, has had a pivotal role in determining the diagnosis.

In a histopathologic examination, reticular abnormality on computed tomography (CT) correlates with fibrosis. The presence of honeycombing on CT corresponds to the presence of honeycombing on biopsy [3, 4]. Ground glass attenuation represents evidence of interstitial inflammation, macrophage airspace filling, patchy fibrosis, or a combination of these conditions [5]. Thus, HRCT can be a noninvasive tool to predict histologic patterns observed in samples obtained by open lung biopsy. However, its recurrent repetition is challenging because of the radiation load and the considerable economic cost.

Because it is easy, noninvasive, rapid, does not require any specific preparation, and has no influence on the patient, pulmonary function test (PFT) is used in patients with IIPs to evaluate the clinical course and severity. However, the correlation between the PFT and a well-known modality for predicting the clinical course and fate of IIPs, HRCT, has not been thoroughly explored. The primary goal of this study is to discover the pulmonary function parameters that have an association with the severity and progression of IIPs, using HRCT as the gold standard.

IIP diagnosis and management have been challenging due to their diverse clinical, radiological, and histopathological features. CT has been the central component and reliable tool for the diagnosis and monitoring of IIPs [5, 6], but the recurrent radiation and cost associated with the imaging make it difficult to use as a routine diagnostic tool, particularly in low-resource countries like Ethiopia. The aim of this research is to investigate the correlation between CT patterns of IIPs and spirometry and to evaluate whether spirometry could be a reliable investigation for predicting the prognosis and outcome of patients with IIPs. The research was conducted in Ethiopia, where the resources for diagnostic imaging are limited.

Spirometry is a noninvasive and affordable investigation, but to the best of our knowledge, there is an absence of data regarding its role in predicting the outcome of patients, and it remains unclear of its usage in predicting the prognosis in patients with IIPs. The proposed study utilized spirometry to evaluate the pulmonary function of patients and correlate the results with the CT patterns of IIPs.

## 2. Materials and Methods

### 2.1. Study Design

An institution-based, retrospective, descriptive, and cross-sectional study design was used.

### 2.2. Study Population and Period

All patients diagnosed with IIPs at the chest clinic of Tikur Anbessa Specialized Hospital (TASH) who underwent both HRCT and spirometry. All patients diagnosed with IIPs who underwent HRCT and spirometry within 3 months of HRCT acquisition at TASH during the past 3 years are included in this study (2020–2023).

### 2.3. Data Collection Tool

The research involved a review of medical records, HRCT images, and spirometry records to collect data on patient demographics, medical history, CT features, and PFT results on a Google form–structured questionnaire. The questionnaire is adopted from the literature. The questionnaire is prepared in the English language and is close-ended.

### 2.4. Inclusion and Exclusion Criteria

#### 2.4.1. Inclusion Criteria

Inclusion criteria included all patients with a diagnosis of IIPs who also had HRCT and spirometry within 3 months of HRCT acquisition at TASH in the last 3 years.

#### 2.4.2. Exclusion Criteria

• Poor-quality HRCT (e.g., significant motion artifact and bubble artifact).
• Inconclusive diagnosis.
• Significant associated emphysematous changes.
• Lack of complete patient record data.

### 2.5. Sampling Technique and Data Collection Procedure

A registry book from the TASH's chest clinic was used to identify 54 patients with the diagnosis of IIPs from February 2020 to February 2023 who underwent pulmonary function testing.

HRCT images and corresponding reports were retrieved from the central picture archiving and communication system (PACS). Axial CT images were obtained at six standardized anatomical levels: the level of the great vessels, aortic arch, tracheal carina, pulmonary hilum, pulmonary venous confluence, and 1 cm above the right hemidiaphragm. Scans were acquired using pulmonary window settings optimized for parenchymal evaluation (mean: 2500–2600 Hounsfield units; window width: 1400–1600 Hounsfield units). This imaging protocol follows the methodology described in previous studies on diffuse lung diseases [1, 7, 8].

The scoring system used was adapted from previously published research, particularly the work of Müller et al. and Biederer et al. A semiquantitative approach was used to assess both the profusion and extent of parenchymal abnormalities, including consolidation, ground-glass opacities (GGOs), reticulation, and honeycombing.

For each of the six HRCT levels, both lungs were divided into a grid composed of 1 cm^2^ squares. Each square was evaluated for parenchymal abnormalities, and the extent of involvement was estimated. The proportion of lung affected at each level was recorded as a percentage. If the area of involvement was less than 10%, the score was estimated to the nearest 1%; otherwise, scoring was rounded to the nearest 5%. The overall lung involvement was then calculated by averaging the percentage scores from the six levels. The final diagnosis was made based on the predominant abnormality seen across the levels, with patterns classified in accordance with standard radiologic definitions.

The overall parenchymal abnormalities, as well as the extent of consolidation, GGO, reticulation, and honeycombing, were assessed. Consolidation was considered present when the opacity hides the underlying vessels, GGO was characterized as an area of haziness with increased attenuation, reticulation was defined as lesions with innumerable interlacing lines, and honeycombing was regarded as clustered cystic airspaces 3 to 10 mm in diameter with layering in the subpleural lungs. The extent of involvement was scored to the nearest 5%, except when less than 10% of the lung is involved; in those cases, estimation was scored to the nearest 1%. The scores of the 6 lung zones were averaged out to obtain a mean score. On each level, the dominant parenchymal abnormality was categorized into the 4 aforementioned abnormalities, and finally, the average dominant parenchymal abnormality was taken as the predominant finding for the specific diagnosis. The results of forced vital capacity (FVC), forced expiratory volume in 1 s (FEV1), peak expiratory flow (PEF), and forced expiratory flow (FEF) of individual patients were collected from the chest clinic database.

### 2.6. Operational Definitions

Percentage of parenchymal abnormalities: When less than 10% of the lung is affected, the estimation was scored to the nearest 1%; otherwise, the extent of involvement was scored to the nearest 5%. 6 predefined levels were taken at the great vessels, aortic arch, tracheal carina, pulmonary hilum, and pulmonary venous confluence, and 1 cm above the right diaphragm. A mean score was calculated by averaging the scores of the 6 lung zones.

Dominant feature: The dominant feature on each level was categorized into the 4 parenchymal abnormalities below, and then the average dominant feature was taken as the predominant feature of the HRCT.

Consolidation: When the underlying vessels are hidden by the opacity.

GGO: Region of increased attenuation and haziness without obscuring the underlying vessels.

Reticulation: Thickening of the interlobular or intralobular septa and appears as several linear opacities that resemble a mesh.

Honeycombing: Between 3 and 10 mm in diameter, clustered cystic air pockets that are typically subpleural and peripheral.

Pulmonary cysts: Round, thin-walled, low-attenuation spaces/lucencies in the lung.

### 2.7. Data Processing and Analysis

To reduce logical errors and design skipping patterns, the collected data were verified for completeness, cleaned, edited, coded, and entered into EpiData Version 3.1. Data were then exported for analysis into SPSS Windows Version 27. Descriptive analysis was done by computing proportions and summary statistics. Then the information is presented by using simple frequencies, summary measures, tables, and figures.

### 2.8. Ethical Consideration

Ethical clearance was obtained from the AAU radiology departments' research and ethics committee. Information was kept confidential by keeping the anonymity of the study subjects. A formal letter of permission and support was written to the internal medicine department, pulmonology unit. Then informed, voluntary, written, and signed consent was obtained from all patients.

## 3. Result

### 3.1. Sociodemographic Characteristics

From February 2020 to February 2023, we identified a total of 54 patients with IIPs who underwent spirometry. The demographic, clinical, and physiologic characteristics of these patients are summarized in Table 1. The median age of patients was 53.9 ± 15.4 years; the age ranges between 27 and 91, and most of the patients were female (64.8%, 35). 8 patients were smokers (14.8%); the mean pack-years among smokers was 7 ± 4.2. Restrictive was the most common PFT diagnosis seen among 64.8% of the patients [9]; 13 patients had normal spirometry.

### 3.2. HRCT and Spirometry Correlation

**Figure 1:**
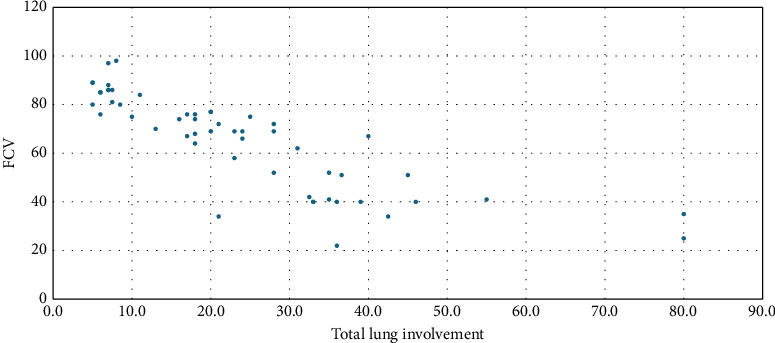
Correlation between the extent of total lung involvement in HRCT and functional vital capacity (FVC) in IIP patients visiting chest clinic, Tikur Anbessa Specialized Hospital, Addis Ababa, Ethiopia, 2020–2023.

Pearson's correlation test was applied, considering a two-tailed *p* value < 0.05 as statistically significant. All the spirometry parameters (FVC, FEV1, PEF, and FEF 25%–75%) and oxygen saturation levels measured by pulse oximetry showed a negative and significant correlation with the extent of lung involvement. The strongest correlation from the parameters seen with FCV and FEV1 is *r* = −0.827 and *r* = −0.789, respectively, with a *p* value of < 0.001 for both, while moderate correlation (*r* = −0.49, *p* value < 0.001) was seen with oxygen saturation. A weak correlation of *r* = −0.39 and *r* = −0.38 with *p* values = 0.003 and 0.005 was identified with PEF and FEF 25%–75%, respectively (Figure 1).

13 patients had a normal spirometer finding; among these patients, the average extent of lung involvement was 6.9 ± 1.3. 76% of the patients had a dominant finding of GGO. The commonest radiological diagnosis was nonspecific interstitial pneumonia (NSIP) (76%), and the next common diagnosis was lymphoid interstitial pneumonia (LIP) (15.4%).

### 3.3. IIP Patterns and HRCT Features

Among IIPs, NSIP was the most common subtype, accounting for 57.4% (*n* = 31), followed by usual interstitial pneumonia (UIP) at 20.4% (*n* = 11) (Table 2). The dominant HRCT findings are summarized in Table 3, with GGO being the most frequent feature, followed by reticulation and honeycombing.

The majority of the patients diagnosed with NSIP were females, 67.7% (21/31), with the dominant feature being GGO at 93% (29/31). The lung involvement shows an apicobasal gradient with the most involved level being the basal level 6 (1 cm above the diaphragm) with a mean value of 38.7% ± 27.1. The mean age for NSIP is 51.6 ± 14.

The predominant PFT finding was restrictive, with the mean FVC and FCV1 values being 66.7% and 67.4%, respectively, from the predicted. Scleroderma/systemic sclerosis was the most prevalent comorbidity for NSIP, present in 9 individuals (17%). All the patients with scleroderma were female with a relatively younger mean age of 43.7 ± 10.7. Mean global extent of involvement on HRCT was 17.8 ± 8.9, which is 45% less than the rest of the patients with NSIP. Mean FVC is also higher (70.3 ± 12).

UIP shows female predominance with 64% [6]. The mean age is relatively older compared to a mean age of 56.7+/15. The results indicate an apicobasal gradient, with the basal lung showing level 6 involvement at 55% and total lung involvement; the mean FVC and FCV1 values were 61.8% and 62.2%, respectively. The most common comorbidity was RA (3/54). The dominant features were honeycombing at 64% (7/54) and reticulation at 36% (4/54). 90% of the patients have a restrictive PFT. In patients without honeycombing and diagnosed with UIP, the CT features included an apicobasal gradient of reticular changes, with the least involved segment being level 1 with 7.5% and level 6 being the most involved with 45%.

All of the 4 LIP-diagnosed patients have associated HIV infection, but no sex predominance was seen on LIP. Patients have an age range of 47–68 and a mean age of 55 ± 9.2. GGO and cystic lesions were the 2 dominant features seen on HRCT. Half of the patients had restrictive spirometry patterns, while the other half had normal spirometry. The mean total lung involvement was 13% ± 6, which is significantly less compared to the NSIP and UIP patterns. LIP also showed basal lung predominance with 23.7% of level 6 and 18.7% of level 5 involvement, contrary to the 12% combined involvement of level 1 and 2. And mean FVC and FEV1 are in the normal range (82 ± 12 and 82.5 ± 14). All the patients have normal CD4 counts with a mean of 627.

## 4. Discussion

The results of this study show that there is a strong negative relationship between FVC1, FEV1, and the global extent of HRCT involvement. Research has shown that HRCT, which accurately describes histopathologic progression in IIPs [2], can also predict histologic patterns and prognosis [3–5]. Similarly, PFT provides valuable insight into lung involvement in IIPs and can be routinely performed to monitor disease progression, with HRCT reserved for cases with clinical uncertainty. FVC, the most reliable PFT parameter, is primarily affected in IIPs due to increased inspiratory load and reduced lung expansion, limiting the air exhaled after a deep breath [10–14]. A similar finding was identified in this study, too. Xaubet and coworkers also described a similar negative, statistically significant, moderate correlation between the extent of lung involvement graded by HRCT and FVC (*r* = −0.51, *p*=0.01) in 39 untreated patients with IPF.

However, there are some studies showing that diffuse lung disease and spirometry do not correlate. Fulmer and colleagues evaluated 23 patients with the diagnosis of IPF. All of the evaluated spirometry values do not correlate with the extent of lung involvement by fibrosis or cellularity [15]. Cherniack and coworkers found similar findings among 96 IPF patients using a semiquantitative histologic scoring system [16]. However, these studies did correlation studies between spirometry and histologic extent of diffuse lung disease, and the disease extent was not evaluated by HRCT, and it is difficult to predict the global extent of involvement solely with histology.

The mean age of patients with IIP in our study was 53.9 years. Similar studies conducted in Ethiopia at Tikur Anbessa Hospital (Addis Ababa) and Ayder Hospital (Mekelle) reported comparable mean ages of 50 and 55, respectively. Research conducted in Paris also revealed a mean age of 55.7 [16]. 70% of the patients were older than 40. The mean age between different types of IIPs ranged from 49 to 62 in studies done on this topic. We observed a female predominance, consistent with studies conducted in Ethiopia and around the world [17–19].

NSIP was the most common IIP diagnosed, with 57.4%, followed by UIP (31.5%), LIP (7.4%), and COP (1.9%). Previous studies from Ethiopia suggest similar findings [17, 20]. These findings, however, disagree with those of other investigations. Collard et al. analyzed published literature on the prevalence and diagnosis of IIPs from 1966 to 2001. The sample sizes of the research they analyzed ranged from 129 to 78, and each study discovered that the UIP was the commonest pattern [21]. Recent studies in Egypt, USA, and France suggest UIP to be the frequently encountered diffuse lung disease pattern [22, 23]. All of the mentioned studies have a lung biopsy, and a confirmatory histopathology test was done for the patients [1, 2, 24]. Flaherty et al. also support this, evaluating the importance of multidisciplinary discussion in reaching a diagnosis for IIPs in their study. The study revealed that radiologists initially diagnosing NSIP changed their diagnosis to UIP after histologic input from a pathologist. In an independent review of HRCT scans, 27 out of 58 cases were diagnosed as NSIP, while only 15 were diagnosed with UIP by cardiothoracic radiologists. However, in a multidisciplinary team setting involving clinicians and pathologists, UIP emerged as the most common IIPs—32 out of 58 cases by radiologist A and 30 by radiologist B—while NSIP diagnoses dropped to 14 and 15 patients, respectively. The absence of a multiciliary approach in all Ethiopian studies may contribute to NSIP emerging as a predominant feature. Furthermore, Melesse and colleagues attributed the predominance of NSIP in their study conducted in Mekelle, Ethiopia, to age variation and genetic differences [17]. Given that this is the third study suggesting NSIP predominance in Ethiopia, further multicentric study with a larger study population encompassing a multidisciplinary team diagnosis is recommended to ascertain the distribution of the IIPs.

Examining individual IIP distribution, 72% of those diagnosed with UIP were aged 50 and above, aligning with findings in other studies [1]. The CT patterns observed in this study, such as apicobasal gradient, predominant basal lung involvement, and honeycombing, are consistent with characteristics noted in previous research [1, 4, 23, 24]. In our study, two-thirds of the patients exhibited honeycombing changes, consistent with multiple studies on the topic. Notably, various studies suggest that honeycombing is a determinant factor for suggesting a UIP pattern with high confidence, demonstrating a sensitivity of 90% and a specificity of 86% [4, 23, 25]. While honeycombing alone on HRCT indicates the presence of UIP with high sensitivity and specificity, the overall UIP pattern of fibrosis needs to be assessed for a confident diagnosis. The most common pattern next to honeycombing was peripheral reticulation with an apicobasal gradient. The most common associated comorbidity with the UIP pattern in our study was RA. This aligns with most studies on this topic, considering UIP to be the most prevalent and potentially most dangerous extra-articular manifestation of RA [26, 27]. In this study, the mean FVC for patients with the UIP pattern was 61.8%. Similar findings from a study conducted at the Mayo Clinic in Rochester, Minnesota, United States, were published, where the mean FVC was 64%. They also found a correlation between the global extent of involvement and the decline in FVC in the UIP pattern.

The mean age of the patients with the NSIP pattern in our study was 51.6, which is lower than the mean age of UIP patients (56.7). Ebner and his colleagues also found NSIP patients were significantly younger compared to UIP in a meta-analysis reviewing twelve studies involving 785 patients (338 NSIP and 447 UIP). The median age was 54.8 years in NSIP cases compared to 59.7 years in UIP cases 30. Other series also found NSIP patients to be decades younger than UIP with a median age of between 40 and 50 [28, 29]. Female predominance was also seen in our study; this was also seen in most studies [30, 31].

The dominant feature on HRCT for patients with NSIP was GGO, which is also described in previous studies related to this topic. In previous series, it was also suggested that having GGO as a predominant pattern of NSIP was used as a distinguishing factor from UIP [28, 30, 32], and it was the only HRCT feature in about one-third of cases. Histologically, this can be explained by either fibrosis, inflammation, or both causing the alveolar walls to expand uniformly. The alveolar septa in cellular NSIP are thickened by lymphocyte and plasma cell infiltrates, while in fibrotic NSIP, collagen buildup is the primary cause of the thickening. These pathological changes can lead to the appearance of GGO on imaging studies [33]. Although apicobasal gradient is described with UIP, our study also revealed that NSIP has basal predominance. However, multiple studies also suggested that NSIP can have basal predominance [2, 24, 34].

The most frequently occurring comorbidities for NSIP were scleroderma/systemic sclerosis (17%) in this study. Several recent reports also found that the commonest rheumatological disease among these patients is scleroderma/systemic sclerosis [9, 34–36]. Although the exact pathology in the lung is not fully understood, systemic sclerosis is an autoimmune rheumatic condition characterized by excessive production and accumulation of collagen in the skin and internal organs [37]. Therefore, fibroblast recruitment/activation of the lung interstitium leads to the accumulation of extracellular matrix and scarring. The mean FVC for NSIP was 66.7%, which is 5% higher than UIP. The reason for this is that UIP has more fibrotic interstitial changes, which hinder the normal expansion of the lungs [36].

All of the patients with the diagnosis of LIP in studies have concomitant HIV infection; this is supported by the fact that idiopathic LIP is rare, and it is highly doubted that it occurs without any systemic autoimmune illness or retroviral infection [2]. Our study's mean age (55) for LIP correlates with other research on the subject, where the mean age varies between 50 and 57 [1, 16, 38, 39]. However, there are studies suggesting the age range could be younger, ranging from 30–50, especially in HIV-infected patients [40]. This difference can be explained by the small sample sizes used in all of the studies on the subject, including this one. We recommend conducting more research with a larger sample size. On HRCT, cysts with GGO were the most frequently observed features. Previous literature also frequently reports these findings [2, 40]. In line with earlier research, the areas of the lung most involved were the basal lungs [39, 41]. Unlike UIP and NSIP, where the commonest spirometry finding was restrictive, the predominant PFT abnormality in LIP was obstructive, which also does not relate with previous studies [40]. Therefore, we recommend further study on the topic with a bigger sample.

## 5. Limitation

A notable limitation of this study is the relatively low sample size, reflecting the inherent challenge of conducting research on a rare disease with low prevalence, such as IIPs. The scarcity of cases may affect the generalizability of the findings to a broader population. Moreover, not having information from biopsies, which is crucial for diagnosing IIPs, is a limitation. This absence could affect how well we understand the disease patterns and might result in misclassifying some cases. Additionally, the exclusion of the DLCO spirometry parameter, utilized in previous research, is due to resource limitations, as this specific test was not conducted in Ethiopia. Future research with larger, multicenter cohorts and the inclusion of biopsy data could address these limitations and provide a more robust foundation for studying IIPs.

## 6. Recommendation

Given the strong negative correlation between FVC1 and FEV1 with the HRCT global extent of involvement, this study recommends incorporating routine PFTs, particularly FVC, as part of the standard monitoring protocol for patients with IIPs. Additionally, we advocate for a multidisciplinary team approach rather than routine radiological reporting when evaluating IIPs, highlighting that incorporating clinicians and histologic confirmation is crucial in atypical UIP and NSIP cases.

## 7. Conclusion

In conclusion, this study highlights a strong negative correlation between FVC1 and FEV1 with the HRCT global extent of involvement in IIPs. The findings support the utility of PFTs, particularly FVC, as valuable tools for monitoring the progression of IIPs, with HRCT serving as a complementary diagnostic method in cases of uncertainty. NSIP was the most common pattern in our study, whereas previous research reported UIP as predominant. This difference may be due to the multidisciplinary approach used in earlier studies, which enabled more accurate identification of atypical UIP cases.

## Figures and Tables

**Table 1:** The sociodemographic, clinical, and physiologic characteristics of IIP patients visiting chest clinic in Tikur Anbessa Specialized Hospital, Addis Ababa, Ethiopia, 2020–2023.

Characteristics	Frequency (*n* = 54)
Age, mean (years) ± SD	53.9 ± 15.4
Sex male/female	
Male	19
Female	35
Smoking history	
Yes	8
No	46
Pack year, mean ± SD	7 ± 4.2
PFT	
FVC% predicted (%), mean ± SD	65.1 ± 19.4
FEV1% predicted (%), mean ± SD	65.46 ± 18.4
FEV1/FVC %, mean ± SD	97.28 ± 11.5
FEF 25%–75%, mean ± SD	82.9 ± 23.5
PEF, mean ± SD	82.87 ± 18.27
PFT diagnosis: normal/restrictive/mixed/obstructive	13/35/2/3
Pulmonary hypertension (> 2.9 cm cutoff)	19
Mean pulmonary diameter, mean ± SD	2.8 ± 0.5
Severity on (echocardiography)	
Mild	6
Moderate	10
Severe	3

**Table 2:** Distribution of IIP patterns in patients visiting chest clinic, Tikur Anbessa Specialized Hospital, Addis Ababa, Ethiopia, 2020–2023.

Patterns	*n* = 54 (%)
UIP	20.4
Probable UIP	11.1
NSIP	57.4
COP	1.9
LIP	7.4
ILD indeterminate	1.9

**Table 3:** Common HRCT features in IIP patients visiting chest clinic in Tikur Anbessa Specialized Hospital, Addis Ababa, Ethiopia, 2020–2023.

HRCT features	*n* = 54
Honeycombing	16.7
Reticulation	20.4
GGO	57.4
Consolidation	1.9
Cystic	3.7

## Data Availability

Raw data supporting this study are available from the corresponding author upon reasonable request.

## Nomenclature

AAU: Addis Ababa University
TASH: Tikur Anbessa Specialized Hospital
IIPs: Idiopathic interstitial pneumonias
ATS: American Thoracic Society
ERS: European Respiratory Society
HRCT: High-resolution computed tomography
PFT: Pulmonary function test
IPF: Idiopathic pulmonary fibrosis
UIP: Usual intestinal pneumonia
NSIP: Nonspecific interstitial pneumonia
COP: Cryptogenic organizing pneumonia
AIP: Acute interstitial pneumonia]
RB-ILD: Respiratory bronchiolitis-associated interstitial lung disease
DIP: Desquamative interstitial pneumonia
LIP: Lymphoid interstitial pneumonia
DLCO: Diffusing capacity for carbon monoxide
FVC: Forced vital capacity
